# Pest Activity and Protection Practices: Four Decades of Transformation in Quebec Apple Orchards

**DOI:** 10.3390/insects12030197

**Published:** 2021-02-25

**Authors:** Gérald Chouinard, Francine Pelletier, Charles Vincent

**Affiliations:** 1 Laboratoire de Production Fruitière Intégrée, Institut de Recherche et de Développement en Agroenvironnement (IRDA), Saint-Bruno-de-Montarville, QC J3V 0G7, Canada; francine.pelletier@irda.qc.ca; 2 Saint-Jean-sur-Richelieu Research and Development Center, Agriculture and Agri-Food Canada, 430 Gouin Blvd., Saint-Jean-sur-Richelieu, QC J3B 3E6, Canada; charles.vincent2@canada.ca

**Keywords:** pesticides, risk indicators, environment, health, fruit damage, monitoring, protection costs

## Abstract

**Simple Summary:**

Commercial orchards from Quebec (Canada) were followed for over 40 years to track patterns of activity of major fruit pests, fruit damage and impacts (environmental and financial) of pesticide applications. Some insects (as expected) were more prevalent in the insecticide-free orchard than in commercial orchards, while others were (surprisingly) more prevalent in commercial orchards or as prevalent in both orchard types. Annual fruit damage in the insecticide-free orchard was extremely high (as much as 98% for a single pest) and mostly from the apple maggot, the plum curculio and the codling moth. In commercial orchards, fruit damage was ten times less important and mostly from the plum curculio, the tarnished plant bug and the oblique-banded leafroller. The number of registered pesticides, the number of applications and the total cost of pesticides gradually increased from 1977 to 2019, while the environmental and health risks followed a downward trend for most pesticide categories. The historical trends presented here were likely modulated by external factors such as the pesticide regulatory framework, the arrival of non-native insects and climate change.

**Abstract:**

A group of commercial orchards from Quebec (Canada) was followed from 1977 to 2019 as part of a project to implement Integrated Pest Management (IPM) practices. Collected data comprised activity of major fruit pests (from monitoring traps), fruit damage at harvest and pesticide applications, from which the annual costs and impacts of protection programs over 42 years were calculated. Activity and fruit damage in commercial orchards were compared to patterns observed in a reference insecticide-free orchard. Some insects (European apple sawfly, codling moth, apple maggot) were more prevalent in the insecticide-free orchard than in commercial orchards, while others were more prevalent in commercial orchards (oblique-banded leafroller) or as prevalent in both orchard types (tarnished plant bug). Annual fruit damage in the insecticide-free orchard was mostly from the apple maggot (up to 98%), the plum curculio (up to 90%) and the codling moth (up to 58%). The average situation was different in commercial orchards, whose damage was mostly from the plum curculio (up to 7.6%), the tarnished plant bug (up to 7.5%) and the oblique-banded leafroller (up to 1.7%). While the number of registered pesticides, the number of applications and the total cost of pesticides gradually increased from 2002 to 2019, the risks incurred, as measured by indicators of environmental and health impacts, followed a downward trend for insecticides and acaricides and varied slightly for fungicides.

## 1. Introduction

Introduced to North America by European settlers as early as 1625, apple (*Malus domestica*) trees were the main cultivated pome fruit for centuries; during this time, there were minimal regulatory and environmental constraints and rudimentary marketing schemes. For the last 40 years, orchardists of northeastern North America have had to cope with numerous arthropod pests and diseases [1] that require several pesticide applications per season to meet current market demand while complying with increasingly stringent external constraints [2,3,4]. This high number of pests can be explained by a number of factors. Abundant rainfall in spring and early summer favors the development of apple scab (*Venturia inaequalis*), a fungal disease that, owing to its potential agronomic and economic risks and impact if unmanaged, is a major driver of protection programs [5]. A perennial plant such as the apple tree also allows pests to establish over a long period of time, and these pests cannot be managed easily by avoidance methods, such as rotations, which are practiced in other crops. Although growers apply few pesticides until the trees start bearing fruit, they have to be economically conservative afterwards (especially during the first few years of production) to recover their investment. Hence, their tolerance to damage incurred by pests and diseases is low during that period. For all these reasons, diseases and pests must be carefully monitored to address the threat they pose before significant and irreversible damage is caused to the crop and to assess the risks they incur.

### 1.1. Protection of Apple Orchards in Quebec, Canada

In addition to diseases, ca. 70 [6] arthropod species may attack cultivated apple trees in Quebec, Canada. These pests can be grouped into three classes according to their economic impact [6]: (1) primary pests that cause economic losses in most orchards; (2) secondary pests that occasionally cause serious damage locally; and (3) minor pests that generally cause negligible damage (Table 1). In the commercial context of North America, 95% damage-free apples at harvest is a tacit benchmark of the success of protection programs [7]. Failure to meet that objective likely implies growers getting lower prices for produced apples. To meet that objective and to produce the quality of fruits expected by consumers, orchardists must implement protection programs that take into account several complex factors. These programs have undergone major changes over the years due to the evolution of the pesticide regulatory framework (e.g., Food Quality Protection Act, discussed below), changes in demand by fresh and processed fruit markets, and considerable advances in science (e.g., entomology, phytopathology) and technologies (e.g., pesticides, traps, pheromones, predictive models), including most notably the advent of the internet and related information handling and delivery technologies. Finally, global factors such as the introduction of invasive species and climate change have presented challenges for existing programs.

Prior to 1950, Quebec orchardists practiced systematic pest management, i.e., a suite of calendar-based interventions irrespective of actual threats to apple trees. Beginning in the 1950s, advances in science produced reliable and efficient monitoring methods such that a first step in streamlining protection programs was achieved. In 1975, the Quebec Ministry of Agriculture created the Apple Pest Warning Service and its Apple Network [8,9], which systematically collected data in a number of orchards representing major apple-producing areas of Quebec [5]. Starting in 1977, participating orchards were monitored as part of an Integrated Pest Management (IPM) project, which later evolved as Integrated Fruit Production (IFP) programs [10,11,12].

Orchardists were asked to carry out their phytosanitary treatments according to monitoring results in their orchards or predictions made by available bioclimatic models [5]. From March to October, entomological and climatic (temperature, rainfall, relative humidity) data obtained from meteorological stations were transmitted weekly to the Quebec Apple Network laboratory (currently the Integrated Fruit Production Laboratory of the Research and Development Institute for the Agri-environment). The data were compiled and transmitted to key persons responsible for informing stakeholders (orchardists and participants) on a weekly basis about the status of apple pests in their region and providing phytosanitary recommendations. Over the years, advances in information technologies made it possible to deliver information more efficiently to stakeholders. This article is based on data collected from 1977 to 2019 in Quebec apple orchards, providing a rare long-term perspective on the evolution of pests, pesticide use and associated impacts. It follows a preliminary study [13] on the historical use of pesticides in Quebec apple orchards that uncovered some of the historical data from 19763–2012.

Over that 42-year period, agronomic practices and pesticide regulations underwent major changes in Canada. In 1977, most apple trees were standard-sized trees with a voluminous canopy (3 m height, 6 m radius) that was difficult to scout thoroughly and to reach completely with sprays. In 2019, most apple trees are grafted on dwarfing or semi-dwarfing rootstocks such that they are grown much more densely and have a canopy amenable to both scouting and spraying.

From 1977 to 2019, in Quebec, the average orchard size almost doubled (6.8–12.6 ha) while the number of apple-producing farms decreased by more than half (2029–463). Similarly, the number of apple trees also more than doubled (1.3M–3.0 M) while the total orchard area was reduced almost by half (10610–5838 ha). In 1977, the vast majority (77%) of apple trees were “McIntosh” grafted on standard-sized rootstocks, while in 2019, this cultivar represented only 35% of trees, which were predominantly grafted on dwarfing (55%) and semi-dwarfing (35%) rootstocks. Over that same 42-year period, total apple production increased by 57% (72–113 metric tons), most of which is sold on the domestic market [14,15,16]. Organic apple production, non-existent in 1977, occupied 188 ha in 2018, i.e., 3.2% of total orchard area [17]. As of 2019, Quebec and Canada had become net importers of fresh and transformed apples [18], leaving room for the apple industry to grow.

A defining event of that period is the Food Quality Protection Act passed by the U.S. Congress and signed by President Bill Clinton in 1996 [19], which established more stringent health-based safety standards on the use of pesticides in the USA. Canada has an independent pesticide registration system that in part reflects U.S. standards. Overall, the array of pesticides registered in the U.S. and Canada changed drastically from 1977 to 2019. Consequently, growers’ use of chemicals reflects changing regulatory constraints, and chemical use cannot be strictly compared between years. Growers must comply with regulations but are ultimately responsible for the choice of the chemicals they use. Nonetheless, it is not in their economic interest to overuse pesticides and they aim to minimize their use so as to avoid risks of economic failure. 

### 1.2. The Apple Industry in Quebec

Quebec’s apple production is concentrated in the regions of Montérégie, Laurentides, Eastern Townships and the Quebec City area, and is the main fruit industry in the province, with a farm gate value of CAD 54 M [15]. Since the first Phytosanitary Strategy was implemented in 1992 by the Government of Quebec [20], apple growers have worked to reduce pesticide use in their orchards. Most growers participate in co-financed IPM scouting services, while others monitor pests and diseases independently. Information collected by the Quebec Apple Network, namely phenology, capture levels of main insects, pesticide use and damage levels and allows stakeholders to assess their situation yearly relative to historical standards. In a 2005 survey, Quebec apple orchards were those in which insecticides and fungicides were applied least intensively among Canadian provinces [21].

## 2. Materials and Methods 

From 1977 to 2019, the Quebec Apple Network monitored participating orchards, which varied from 5 to 12 over the years. Orchards that ceased participation for various reasons were discarded from the dataset presented below. One insecticide-free (reference) orchard was included to provide baseline data on pest population levels and fruit damage in the absence of treatments. The orchards varied from 1 to 5 ha, and all of them were homogenous, established plantings and belonged to an orchard enterprise from a given region. No incentive was offered to growers to participate other than first-hand access to collected data, which was sufficient to provide long-term participation for the vast majority of them. From 1977 to 2001 (hereafter Phase 1), the insecticide-free orchard was located at the Experimental Farm of Agriculture and Agri-Food Canada in Frelighsburg. From 2002 to 2019 (hereafter Phase 2), the insecticide-free orchard was located at the IRDA experimental site at Saint-Bruno.

From March to October, meteorological data (i.e., temperature, rainfall and relative humidity) were collected weekly by meteorological stations positioned in participating orchards. Phenological stages of the apple trees were noted and several pests were monitored weekly (Table 1) using recommended methods [6,22] based on research conducted in Quebec and elsewhere. Some pests were monitored occasionally when their economic importance was of concern to stakeholders. Damage was assessed every year in each orchard by examining fruit at harvest. Participating growers transmitted their seasonal treatment programs to the Apple Network.

### 2.1. Monitoring Apple Pests

**Figure 1:**
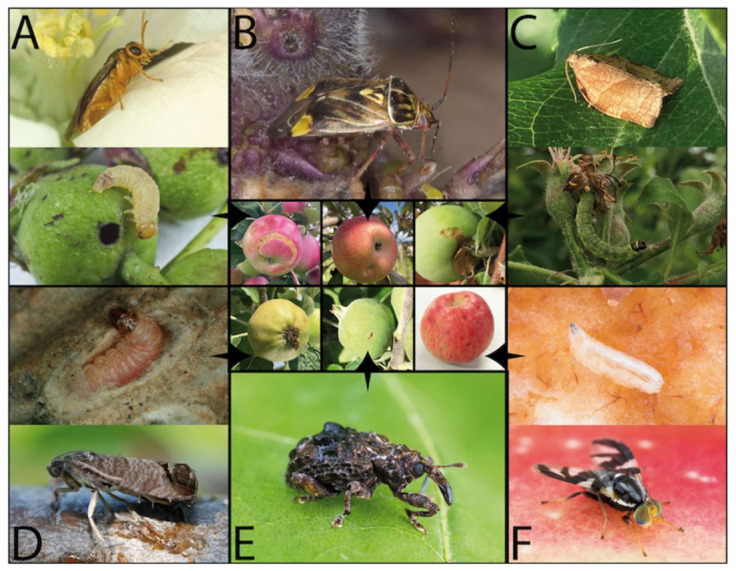
(**A**) European apple sawfly (adult and larva); (**B**) tarnished plant bug (adult); (**C**) oblique-banded leafroller (adult and larva); (**D**) codling moth (adult and larva); (**E**) plum curculio (adult); (**F**) apple maggot (adult and larva); at the centre: fruit damage caused by these insects. Photos: F. Pelletier (1**A**, damage; 1**B**, damage; 1**C**, adult and larva; 1**D**, damage); F. Vanoosthuyse (1**B**, adult; **1C**, damage; 1**D**, adult and larva); A. Charbonneau (1**E**, damage); IRIIS (www.iriisphytoprotection.qc.ca, accessed on 25 February 2021) for other pictures.

Four monitoring tools were used. Adults of the apple sawfly (*Hoplocampa testudinea* Klug) (Figure 1A) [23] and the tarnished plant bug (*Lygus lineolaris* P. de B.) (Figure 1B) [24,25] were caught with white sticky traps. Adults of fruit-feeding lepidopterans (Figure 1C and Figure 1D) were caught with specifically-lured pheromone traps, either sticky (Pherocon 1C) (1977–2001) or non-sticky (Multi-Pher I) (2002–2019) [26,27,28,29]. Adults of the plum curculio (*Conotrachelus nenuphar* (Herbst) (Figure 1E) were monitored with black pyramidal traps [30]. Adults of the apple maggot (*Rhagoletis pomonella* (Walsh) (Figure 1F) were trapped with ammonia-baited yellow sticky traps (Pherocon AM) until 1991, when they were replaced by the new standard, i.e., unbaited sticky-coated red spheres [31,32]. Despite being considered a secondary pest, the European apple sawfly was included because of concerns regarding its expansion at the beginning of the study.

In each orchard, two pheromone traps or four white sticky traps or sticky red spheres were positioned in uniform blocks of 5 ha. Pheromone traps were positioned inside apple tree canopies at 1.5 m above ground level. White sticky traps were positioned at ca. 15 cm from a fruit cluster at either 0.75 (tarnished plant bug) or 1.5 m (apple sawfly) above ground level. Sticky red spheres were positioned at 15 cm from a fruit cluster and at 1.5 m above ground level. All traps were examined weekly from April 1 until the end of the season (September–October). Pheromone dispensers were refreshed monthly. Sticky red spheres were cleaned weekly, while white sticky traps were replaced when needed, their stickum being refreshed at least monthly.

### 2.2. Damage Assessment at Harvest

Each year during the last week of August (ca. 10–14 days prior to the beginning of harvest), 500–1000 fruit per participating orchard were examined randomly to assess the performance of protection programs. All fruits were examined visually, and damage (insect, diseases, physiological problems) was tallied. 

### 2.3. Protection Programs

In each orchard, the number of applications, the formulations and the dosages of sprayed insecticides, acaricides and fungicides (including antibiotics used against some diseases) were transmitted by participating orchardists. The costs of pesticide treatments (Table S1) (phytosanitary products only–other costs such as manpower and machinery excluded) are reported in constant (base 2019) CAD [33]. The impact of treatments on the environment and health were estimated by risk indicators [34,35]. The Environmental Impact Quotient (EIQ) [36] developed at Cornell University was first used (Phase 1: 1977–2001) because of its simplicity, publicly accessible database (mainly constructed from EXTOXNET, SELECTV, CENET CHEM-NEWS, USDA Soil and Pesticides Database) and adaptability for estimating the risks associated with pesticides used in apple orchards [37,38]. The EIQ assigns a variable value to the following parameters: chronic and skin toxicity, half-life on leaves and soil, mode of action and absorption, leaching potential, bio-accumulation and toxicity to mammals, fishes, birds, bees and natural enemies. The date of treatment is also taken into account by the orchard variant of the EIQ [37]. Starting in 2002 (Phase 2), the Quebec pesticide risk indicator (IRPeQ) [39] was used as it became available, and a few years later became the standard for the Government of Quebec. It was not possible to use the same indicator over the whole 42-year period, since the oldest pesticides were absent from the new IRPeQ database and inversely, the newest pesticides were absent from the EIQ database, which was no longer updated. The IRPeQ features two indexes: one for environmental risk (IRE) and one for health risk (IRS). The parameters, database and relevant information are periodically updated by the Government of Quebec and are available at https://www.sagepesticides.qc.ca/Information/IndicesRisques (accessed on 24 November 2020).

To calculate the annual impact of pesticide treatment programs, the values associated with each active ingredient were multiplied by the percentage of the active ingredient in a given formulation and the dose applied, as follows:

**∑**{(RI_i_) × AI_i_ × R_i_}

where:

RI_i_: value of risk indicator of pesticide i

AI_i_: % active ingredient in formulation of pesticide i

R_i_: dose (quantity per ha) in formulation of pesticide i applied Temporal trends were established by linear regression (indices of risk vs. year for all sites) and reported for significant values of r (*p* 0.05). 

## 3. Results and Discussion

### 3.1. Pest Activity 

Pest activity is presented as weekly mean captures per trap, and the annual fluctuations (due to inherent variability of biotic and abiotic conditions and of pesticide use) can be seen in Figure S2.

#### 3.1.1. Tarnished Plant Bug (*Lygus lineolaris—*Miridae)

**Figure 2:**
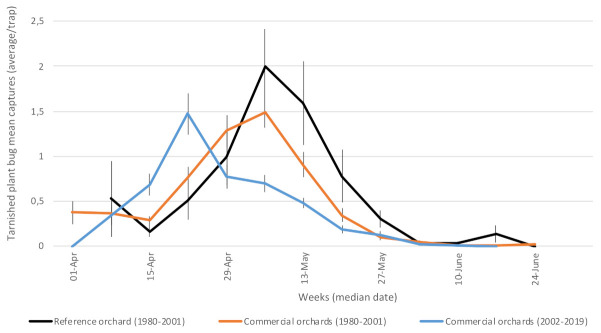
Average captures of tarnished plant bug adults in participating orchards. Reference (insecticide-free) orchard located at Frelighsburg, Qc (Agriculture and Agri-Food Canada, 1980–2001). Commercial orchards located at Franklin, Hemmingford, Oka, Rougemont and Saint-Paul-d’Abbotsford. Vertical bars represent standard error.

Overwintered adults resume their activities in early spring by feeding on buds (Figure 2). They can feed on fruitlets, leaving depressions of various sizes on the epidermis, thus affecting fruit quality at harvest [40]. Prediction of their abundance from bud break to fruit set is difficult and unamenable to determining the risk incurred and optimal timing of intervention. Thus, tarnished plant bug is frequently a concern in orchards where problems have been documented.

#### 3.1.2. European Apple Sawfly (*Hoplocampa testudinea—*Tenthredinidae)

**Figure 3:**
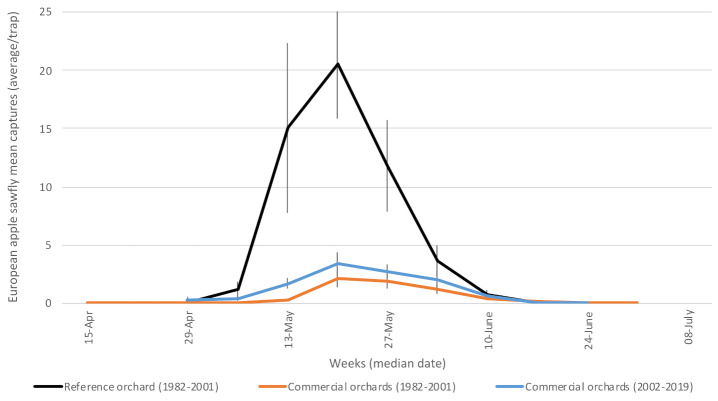
Average captures of European apple sawfly adults in participating orchards. Reference (insecticide-free) orchard located at Frelighsburg, Qc (Agriculture and Agri-Food Canada, 1982–2001). Commercial orchards located at Franklin, Hemmingford, Oka, Rougemont and Saint-Paul-d’Abbotsford. Vertical bars represent standard error.

Paradis [41] first reported the apple sawfly in Canada in 1980 from specimens captured in Hemmingford, Qc. Adults were active (Figure 3) from the pink stage [42] until a few days after the fruit set, as reported in previous studies [43]. Because entomophilous pollination is critical for apple production, applications of insecticides during bloom are proscribed, thus reducing the optimal window to treat with adulticides. 

#### 3.1.3. Plum Curculio (*Conotrachelus nenuphar*—Curculionidae)

**Figure 4:**
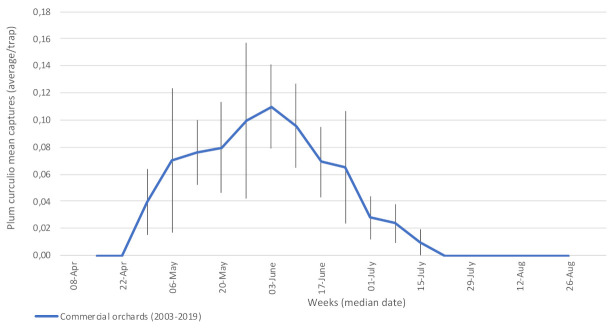
Average captures of plum curculio adults in participating orchards, 2003–2019. Commercial orchards located at Franklin, Hemmingford, Henryville, Rougemont, Saint-Bruno-de-Montarville and Saint-Paul-d’Abbotsford. Vertical bars represent standard error.

Adults can cause four types of damage on fruits: (1) half-moon scars made in the early season by ovipositing females; (2) damage caused by larvae feeding on internal tissues and pips; (3) June drop of fruitlets attacked; and (4) circular damage of the epidermis caused by feeding adults in August. Monitoring of this key pest of pome and stone fruit of Eastern North America is still tedious and unreliable. Dark wooden pyramids [30] were used in participating orchards starting in 2003, and they yielded relatively less reliable results than the methods used for other insect pests. The first captures of adults occurred in mid-May at the pink stage (Figure 4), a period coinciding with the emergence of overwintered adults from the soil and foraging towards their hosts. They reached a peak at the beginning of June (fruit set stage) as females began to oviposit. A single female may lay up to 200 eggs (most often one egg per fruit) in the weeks following fruit set, making this insect an important pest to monitor until mid-July [44]. 

#### 3.1.4. Oblique-Banded Leafroller (*Choristoneura rosaceana*—Tortricidae)

As in other leafroller pests of apple trees, feeding larvae cause damage to leaves and fruits [45]. As early instars and damage to fruit are often difficult to distinguish in field conditions, monitoring of adults is necessary to document risks requiring interventions. 

**Figure 5:**
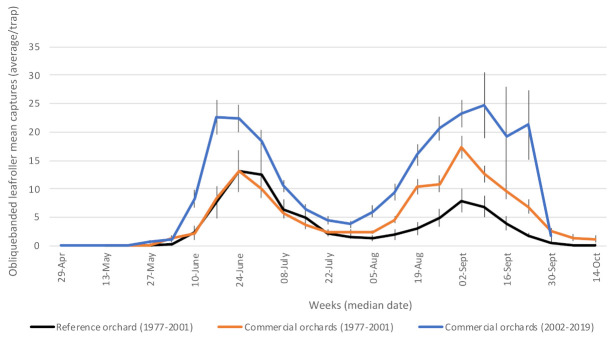
Average captures of adult oblique-banded leafroller in participating orchards. Reference (insecticide-free) orchard located at Frelighsburg, Qc (Agriculture and Agri-Food Canada, 1977–2001). Commercial orchards located at Franklin, Hemmingford, Oka, Rougemont and Saint-Paul-d’Abbotsford. Vertical bars represent standard error.

Overwintered larvae complete their development in spring, and early flying adults are caught starting mid-June (Figure 5). Larvae of the first (overwintered) generation feed on developing buds and fruits, while larvae of the second (summer) generation feed on the epidermis of fruits as harvest approaches. Average captures of adults of the summer generation were greater in the second phase of the study than in the first. This increase cannot be attributed to the different monitoring methods used in phase 1 vs. phase 2, since the new method was chosen for its ease of use—it is not more sensitive than the previous one [27,28]. This can also be appreciated by looking at annual records of trap captures (Figure S2).

#### 3.1.5. Codling Moth (*Cydia pomonella—*Tortricidae)

**Figure 6:**
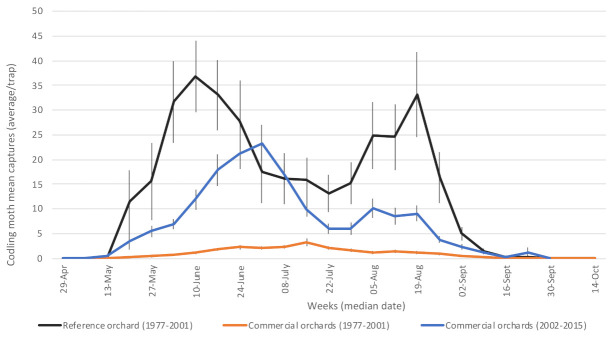
Average captures of codling moth adults in participating orchards. Reference (insecticide-free) orchard located at Frelighsburg, Qc (Agriculture and Agri-Food Canada, 1977–2001). Commercial orchards located at Franklin, Hemmingford, Oka, Rougemont and Saint-Paul-d’Abbotsford. Vertical bars represent standard error.

Widely distributed in Quebec apple orchards and worldwide, this non-native pest completed two generations per season in participating orchards (Figure 6). Codling moth overwinters as larvae. Larvae of the two generations attack fruits, frequently leaving frass at the entry point, thus causing commercial losses. As neonates penetrate fruit a few hours after their emergence, monitoring adults is a key strategy to reliably assess risk (based on the appearance of damage on fruits) and thus optimize time interventions. The first adults were caught on average in mid-May in the studied commercial orchards. During phase 1, average captures in commercial orchards were kept in check by insecticides and remained very low until the end of May (Figure 6). They were, however, much more important during phase 2, and as for the oblique-banded leafroller, this cannot be attributed to the change in the monitoring method, since (a) the new method was chosen for its ease-of-use despite being less sensitive than the previous one [27,28]; and (b) fruit damage was similarly more important in phase 2 than in phase 1 (Table 2). First-generation captures also peaked earlier in the pesticide-free orchard than in commercial orchards, even though climatically similar orchards were compared. 

The two generations were sometimes hard to distinguish because of delayed emergence of adults, overlapping ovipositing periods and low population levels on some sites. Moreover, the second generation was almost non-apparent in commercial orchards, which is difficult to explain with confidence. One of the most plausible causes is related to variable effects of insecticides in commercial orchards, notably the induction of early diapause in summer larvae [46,47].

#### 3.1.6. Apple Maggot (*Rhagoletis pomonella—*Tephritidae)

**Figure 7:**
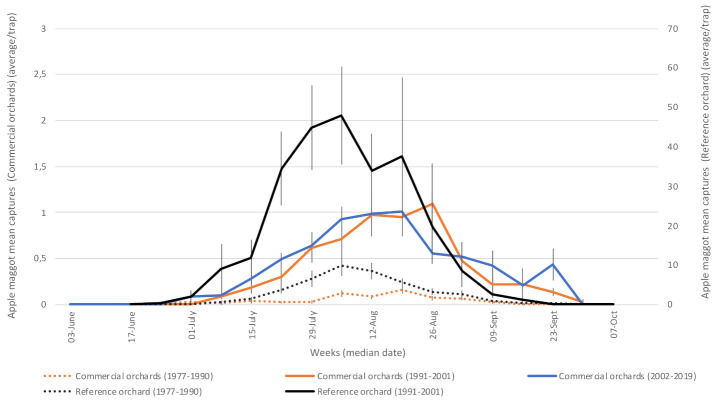
Average captures of adult apple maggot in participating orchards. Reference (insecticide-free) orchard located at Frelighsburg, Qc (Agriculture and Agri-Food Canada, 1977–2001). Commercial orchards located at Franklin, Hemmingford, Oka, Rougemont and Saint-Paul-d’Abbotsford. Vertical bars represent standard error.

This species completed one generation per year, following emergence from the ground where they had overwintered as pupae for the last 1–3 summers [48]. The first adults were caught at the end of June (Figure 7) as females start foraging to oviposit their eggs in the epidermis of developing fruits. Adults were active until harvest. Apple maggot damage is not apparent externally, so it is not until fruits are cut open at harvest that browning internal tissues are revealed [49]. Damage to fruit can be prevented by adulticidal sprays timed by monitoring adults [3] (see also Protection programs below). Captures were much lower when traps consisted of baited yellow sticky panels (Figure 7: dotted lines) than in later years, when traps consisted of unbaited red sticky spheres. The increase in captures in phase 2 could be due to an increase in populations and/or to the higher sensitivity of the new monitoring technique [31], but the former is the most probable explanation. Looking at short-term (3-year) captures before and after trap changes, we measured on average a 5-fold increase in cumulative trap catches following the adoption of the red spheres. We similarly measured a 10-fold increase in the long term, i.e., when comparing average captures in phase 1 with average captures afterwards.

#### 3.1.7. Other Fruit Pests

In addition to the species discussed above, monitoring in participating orchards made it possible to detect or partially determine the activities of apple scab (*Venturia inaequalis* (Cke.) Wint. (ascospore ejections measured each year in spring), the lesser appleworm (*Grapholita prunivora* (Walsh) Tortricidae) (monitored annually from 2010 to 2016), the pale apple leafroller (*Pseudexentera mali* Free Tortricidae) (monitored from 1977 until to 1992), the fruit-tree leafroller (*Archips argyrospila* (Wlk.) Tortricidae) (monitored from 1977 until to 1993), the red-banded leafroller (*Argyrotaenia velutinana* (Wlk.) Tortricidae) (only periodically monitored), the oriental fruit moth (*Grapholita molesta* Busck Tortricidae) (detected annually [50] in Quebec orchards since 2006), the brown marmorated stink bug (*Halyomorpha halys* (Stal) Pentatomidae) (an invasive species detected annually in some Quebec localities [51] since 2016) and *Atractotomus mali* (Miridae) (previously reported in Ontario, found for the first time in Quebec orchards [52] in 2015).

### 3.2. Fruit Damage

Each year at harvest, the examination of fruits in the insecticide-free orchard made it possible to determine the relative potential of pests when left unchecked (Table 2). In the absence of insecticide treatments, average (1977–2019) damage to fruit at harvest was 50% (apple maggot), 48% (plum curculio), 20% (codling moth) and 21% (phytophagous mirids, including 6% caused by the tarnished plant bug). The apple scab, a major disease of apple trees grown in continental humid areas, affected 100% of fruit in the absence of fungicide treatments. For this reason, the insecticide-free orchard was treated with fungicides occasionally and cannot be considered a fungicide-free orchard. Leafrollers caused over 50% of fruit damage over several years (e.g., 70% in 2001; average 26%), and damage from apple sawfly often exceeded 7% between 1977 and 2019 (e.g., 9 and 8% in 2001 and 2002), as reported previously [21]. Altogether, insect pests exerted considerable pressure in the insecticide-free orchard.

Due to the use of pesticides, the estimated damage to fruit at harvest in commercial orchards differed markedly from that in the insecticide-free orchard. In commercial orchards, the most damaging pests were tarnished plant bug (1.7% fruit damage at harvest), apple scab (1.4%) and leafrollers (1.1%). At 0.6, 0.5 and 0.5%, respectively, other phytophagous mirids, plum curculio and the apple sawfly ranked, respectively, the 4th, 5th and 6th most damaging pests. 

Despite capture levels being similar in commercial and insecticide-free orchards, the relatively higher tarnished plant bug damage in commercial orchards (Figure 2) may be explained as follows. First, orchardists must decide whether to treat tarnished plant bug pre-(preventing damage) or post-bloom, while the available monitoring method is often unreliable. Second, there are few registered and efficient insecticides against this pest. Pyrethroids are generally recognized as efficient, but they may negatively impact natural enemies that play an important role in orchard IPM [4].

The oblique-banded leafroller, whose summer larvae characteristically eat the fruit epidermis near the peduncle [45], was the main leafroller pest. Insecticide treatments targeted against overwintered and summer generations larvae often fail to provide a satisfactory control level, notably when populations have developed resistance to organophosphates, as reported in Quebec [53,54] and in New York State [55]. In the commercial orchard located in the Laurentides region (where the previously mentioned organophosphate resistant populations were found), 5% fruit damage at harvest was attributed to this species in 1984, 1985, 1997 and 1998, while other participating orchards experienced an average of 0.05% (0–0.2%) fruit damage. Reissig (1978) [56] also reported important fruit damage by the oblique-banded leafroller in orchards of the northeastern USA a few years earlier. 

Even when limited to peripheral rows, organophosphate treatments targeted at plum curculio adults generally protected fruit from damage [57,58,59]. Broad-spectrum insecticides sprayed as adulticides against the apple maggot ensured not only satisfactory control level of this pest, but also of codling moth between 1977 and 2001 (Table 2). Since 2002, specific treatments against the codling moth were required in most orchards for reasons that remain to be elucidated. Amongst suspected reasons are the development of resistance to some insecticides, the inefficacy of some registered insecticides and climatic changes affecting the development of the insect.

The low levels of damage caused by the apple sawfly may be explained by the efficacy of insecticide treatments against larvae at the pink stage and adults at petal fall [60]. In some orchards, larval parasitism by *Lathrolestes ensator* (Ichneumonidae) may have been a contributing factor [61,62,63].

### 3.3. Protection Programs

Since 1977, IPM practiced in Quebec apple orchards has relied on a rational choice of pesticides and the use of low-risk alternatives. In time, the programs have become increasingly complex, evolving from relying on broad-spectrum pesticides sprayed on a calendar-basis to relying on sprays of selective pesticides driven by monitoring results. It is thus necessary to be cautious when analyzing trends in pesticide use, as stressed in other published studies [64]. First, several disparities relevant to abiotic (e.g., rainfall, average temperatures), biotic (i.e., key arthropod pests), agronomic (i.e., main apple cultivars to be protected) and regulatory (i.e., array of pesticides registered at a given time) factors preclude rigorous comparisons across major apple-producing regions of the world. Second, an increase in pesticide use does not necessarily imply IPM failure, because all of these factors may vary within the same region within a multi-year time frame.

During the last four decades, the number of active ingredients available globally rose from 200 to nearly 600 [65], at a rate of over 100 new ingredients per decade, except for the last decade, when this rate dropped by more than 50%. However, half of the pesticides available in the early 1990s have since been banned in Europe, and new compounds have gradually replaced the more hazardous ones that were introduced during the four previous decades [65]. 

**Figure 8:**
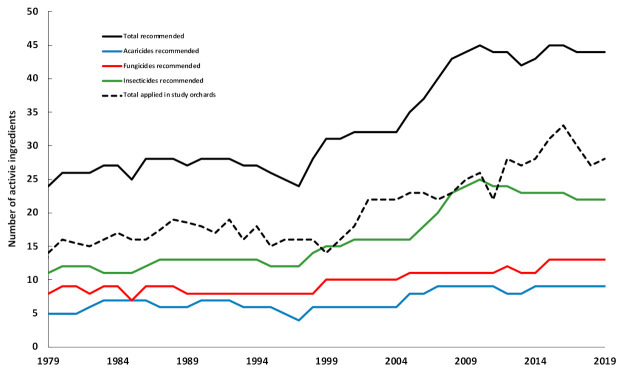
Number of active ingredients of pesticides recommended for use in apple orchards from 1977 to 2019. Compiled from recommendations by the Quebec Government (references: see Table S3).

A somewhat similar pattern was observed in our study. In 1977 and 2019, respectively 24 and 44 pesticides were available for use in apple orchards in Canada, and 20 were banned over the course of this period (Figure 8; Table S3). However, as recently registered pesticides are typically less persistent and more selective, more applications are required annually to achieve commercially acceptable control levels. In our study, the number of applications explained only a minor fraction of the overall variation in the total impact of pesticides used annually in each orchard, depending on the indicator and the period (EIQ 1977–2001: 19%; IRPeQ, 2002–2019: 42–45%). Consequently, the number of sprays and their doses are considered unreliable indicators of their environmental impacts on apple orchards [37], as reported for agriculture in general [66]. The number of sprays per season may nevertheless be useful to calculate the costs of control measures (Table 3).

With a yearly average of ca. 13 applications, fungicides accounted for over 50% of pesticide costs in our study, i.e., CAD 785 per season out of a total CAD 1415 for pesticides. The number of applications and the average annual costs of fungicides applied during phase 2 (CAD 1006) were much higher than during phase 1 (CAD 61). This can be explained by a significant increase in both the cost per application of fungicide and in the annual number of applications (see Table S4). Apple scab was the driver of fungicide programs and accounted for 90% of applications in this category. Factors such as prevailing climatic conditions, cultivars (e.g., McIntosh is vulnerable to apple scab infections) and active ingredients currently registered constitute constraints to further reduction in fungicide use. Alternatives with a low environmental impact, such as potassium bicarbonate and apple scab-resistant cultivars, are promising approaches, some benefiting from governmental financial support to mitigate costs and impacts of protection programs against this disease.

In participating orchards, a seasonal average of 3.9 insecticide and 1.6 acaricide treatments were also applied from 1977 to 2019. Insecticides were used at critical phenological stages (pink and fruit set) or other moments triggered by the results of pest monitoring, according to recommended methods and thresholds [2,67,68,69,70,71,72,73,74,75]. The costs of these treatments per ha varied depending on the choice of products, doses applied and surfaces treated. Globally, although the total number of applications targeting arthropods (acaricides and insecticides) was similar from 2002 to 2019 (phase 2) versus 1977 to 2001 (phase 1), their costs were much higher during phase 2. The increase in total costs was caused by a significant increase in the cost per application for both insecticides and acaricides, and a significant increase in the annual number of applications of insecticides (see Table S4).

A frequently given reason to justify the increase in costs of arthropod management is the development of resistant populations, as documented in several apple-producing regions of North America, including Quebec, notably in phytophagous mites [76,77,78], the oblique-banded leafroller [54,79,80] and the codling moth [81,82]. The situation is less problematic for other pests, such as the plum curculio, as treatments of border rows [57,58,59] now increasingly replace full block treatments, thus reducing pesticide use on a per ha basis for that pest. Bioclimatic models are also increasingly used to predict periods of high activity [83,84,85]. Thus far, plum curculio has not been reported to be insecticide-resistant.

**Figure 9:**
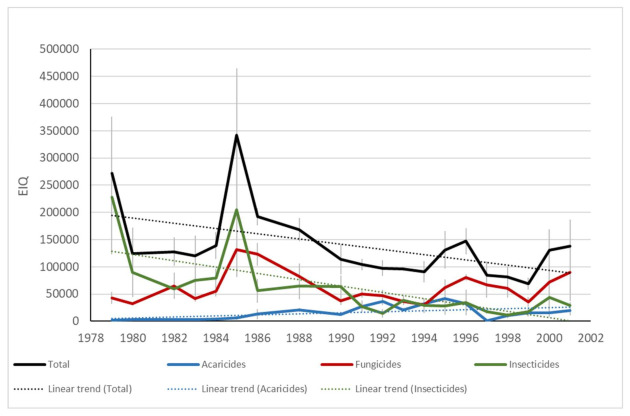
Impact of pesticides as expressed by the Environmental Impact Quotient (EIQ) and estimated annually from treatments applied in commercial orchards from 1979 to 2001. Orchards located at Hemmingford, Franklin and Oka. Vertical bars represent standard error. Only significant trends (*p* 0.05) are shown.

**Figure 10:**
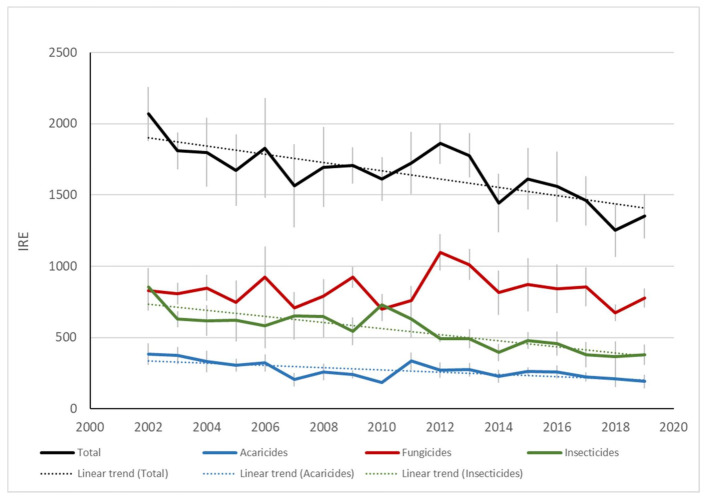
Impact of pesticides as expressed by the Quebec environmental risk indicator (IRE) and estimated annually from treatments applied in commercial orchards from 2002 to 2019 (phase 2). Orchards located at Compton, Dunham, Franklin, Hemmingford, Oka, Sainte-Famille and Saint-Joseph. Vertical bars represent standard error. Only significant trends (*p* 0.05) are shown.

**Figure 11:**
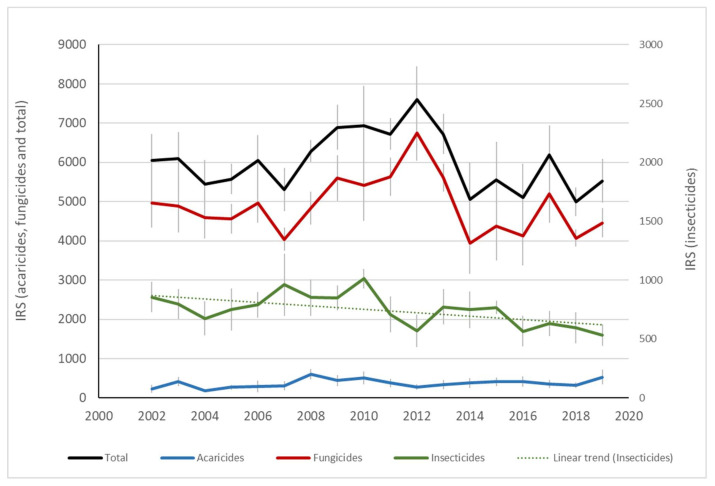
Impact of pesticides as expressed by the Quebec health risk indicator (IRS) and estimated annually from treatments applied in commercial orchards from 2002 to 2019 (phase 2). Orchards located at Compton, Dunham, Franklin, Hemmingford, Oka, Sainte-Famille and Saint-Joseph. Vertical bars represent standard error. Only significant trends (*p* 0.05) are shown.

The analysis of risk indicators showed that the overall impact of treatment programs followed a downward trend between 1979 and 2001 (phase 1), mainly due to a decrease in the overall impact of insecticides and acaricides (Figure 9). This trend was also observed between 2002 and 2019 (phase 2) for insecticides and acaricides, while the overall impact of pesticides used against diseases did not follow a definite trend over the course of the study (Figure 10 and Figure 11). While all our indicators (health and environment) suggested that more progress was achieved during phase 1 than phase 2, agronomic differences and the use of different indicators preclude robust comparisons between these two periods. In addition, in the latter period, climate change brought higher average temperatures, thereby favoring the development of pests and diseases [86], while an increase in global trade contributed to the advent of invasive pests.

Perusal of IRPeQ indicators revealed that, at the beginning of phase 2 (i.e., 2002), most of the environmental impacts were associated with arthropod control (i.e., insecticide and acaricide use), while most of the health impacts were associated with disease control (fungicide and bactericide use) (Figure 10 and Figure 11). However, since 2012, disease control has been responsible for most of both health and environmental impacts, the downward trend for those impacts being observed only for insecticide and acaricide use.

**Figure 12:**
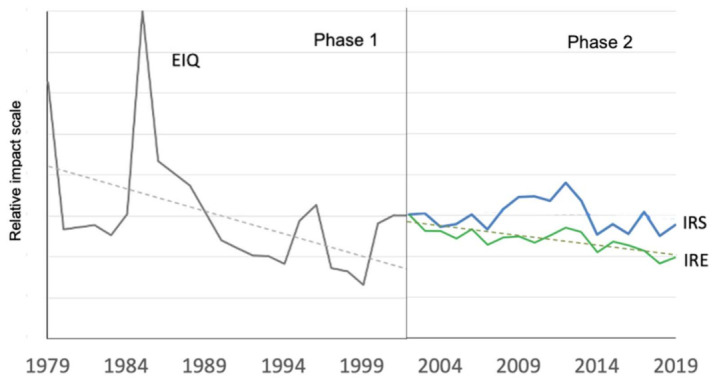
Summarized view of pesticide impacts based on annual treatments applied in commercial orchards from 1979 to 2019. Full lines: mean values; dotted lines: significant (*p* 0.05) linear trends. Orchards located at Franklin, Hemmingford and Oka (phase 1) and at Compton, Dunham, Franklin, Hemmingford, Oka, Sainte-Famille and Saint-Joseph (phase 2). Vertical bars represent standard error. The starting point of the IRS/IRE curves (2002) has been appended to the endpoint of the EIQ curve (2001). All lines and curves are isoscaled.

Overall, environmental impacts decreased more than health impacts during phase 2 (Figure 12). However, the calculated health impacts did not consider prescribed measures such as personal protective equipment, safety features of sprayers and proximity of the treated areas to inhabited or other sensitive areas. Over the years, growers became increasingly aware of the importance of such measures to mitigate their exposure to pesticides, but potential gains due to increased compliance to such prescribed measures have not been fully determined.

A number of outlier points (peaks) in Figure 12 raise questions about their significance. With the exception of 1996 (when higher impact was significantly (*p* 0.05) correlated with higher rainfall and with increased use of metiram fungicide), no significant correlation was found between cumulative rainfall during the primary scab infection period (May–June) and impact quotients. Looking at individual spray programs, however, we observed that the occasional use of high-impact insecticides seemed the most probable reason explaining other peaks observed, for example, in 1979 and 1985, when lead arsenate and demeton were used, respectively, by some growers. Various reasons (marketing, novelty, supply shortage for usual products, etc.) can trigger individual choices.

Except for one study carried out in the Trentino region of Italy [38], we failed to find scientific publications systematically reporting the environmental impacts of pesticide treatments in commercial apple orchards. Reports on pesticide use are available for fruit grown in the USA and a few other countries, but at best they present annual amounts (in kg of active ingredients per surface unit) and/or number of applications [87,88,89,90,91]. The latter indicator gives an equal importance to 1 kg of passive products such as mineral oil and to 1 kg of very potent neurotoxic products such as azinphos-methyl which, as previously stated, can be misleading. Similar biases affect the number of applications; for example, when one methomyl application is replaced by two *Bacillus thuringiensis* applications to control the oblique-banded leafroller, the number of applications doubles and the weight (in kg of active ingredients applied) more than doubles, while environmental and health indicators actually dramatically decrease [37]. Although the relatively small number of orchards surveyed in our study could hardly be considered representative of pesticide *use* throughout northeastern North America, the temporal trends documented here are, in our view, valuable indicators of the *evolution* of plant protection practices in Quebec apple orchards.

## 4. Conclusions

The monitoring program conducted in participating orchards of the Quebec apple network from 1977 to 2019 made it possible to measure and follow the evolution of pest activity, associated protection costs and crop damage in the absence and presence of protection programs. It showed the benefits of implementing IPM and IFP programs, in terms of environmental and health impacts. By choosing, dosing and applying pesticides based on (1) measured levels of pest populations as a proxy for phytosanitary risk incurred in the absence of treatments and (2) pesticide selectivity in terms of environmental and health impacts, apple growers reduced overall pesticide impact as measured by the chosen risk indicators, but failed to contain the increase of their protection costs. Other benefits of IFP, such as improving the social acceptability and sustainability of the apple industry, although more difficult to quantify, still support IFP development. For example, reduced pesticide use likely limited the development of resistance, while meeting the demands of consumers for fruits produced with minimal pesticidal inputs. 

Fruit growers know that the fight against pests is never-ending, driven by constant changes in climate, market conditions, beneficial organisms, pesticides and other factors. The challenges are numerous [7], and research remains essential in order to improve the efficiency, accessibility, durability and safety of current control methods, as well as to develop alternatives. Research must be proactive to avoid setbacks and ensure the availability of apples appreciated by consumers and produced responsibly and in accordance with food industry expectations. The innovation chain must therefore allow the transmission of agri-environmental breakthroughs, from the laboratory to the orchard, so that their benefits can be fully realized. Many future successes are likely to be the result of discoveries that have been in this process of transfer to the farm for some time. The large-scale adoption of mating disruption against codling moth is a striking example: although the first studies proving its effectiveness were completed in 1976 in Europe [92,93], in 1986 in the USA [94], and in 1993 in Quebec [95], several more decades passed before its adoption in northeastern North America [96], in many cases following the availability of financial support from governments [97].

There are many other alternatives to chemical control for apple pests, such as the use of an attract-and-kill strategy against apple maggot [98], classical biological control against European apple sawfly [61,62,63], entomopathogenous nematodes against the plum curculio [99,100], granulosis virus against codling moth [101,102], leaf shredding against apple scab and leafminers [103], exclusion techniques such as cellulose sheets to manage apple sawfly and plum curculio [104] and netting against numerous pests, including invasive ones like the brown marmorated stink bug [50,105,106]. The implementation and integration of these and other innovative techniques into existing programs remain to be achieved and would offer sustainable protection programs over the coming decades. The cost of those alternatives however is generally higher than the cost of pesticides. Implementation could be accelerated through financial incentives, such as green subsidies and/or pesticide taxes, which have been introduced in France, Denmark, Norway and Sweden, and, when high enough, have proven to be effective in significantly reducing the application of pesticides and their associated risks [107].

## Figures and Tables

**Table 1:** Diseases and arthropod pests monitored by the Quebec Apple Network from 1977 to 2019.

Common Name	Latin Name	Family (Part Attacked ^1^)
**Primary pests**		
Plum curculio	*Conotrachelus nenuphar* (Hbst.)	Curculionidae (F)
Codling moth ^2^	*Cydia pomonella* (L.)	Tortricidae (F)
Fire blight	*Erwinia amylovora* (Burrill) Winslow et al.	Enterobacteriaceae (F,L,W)
Apple maggot	*Rhagoletis pomonella* (Walsh)	Tephritidae (F)
Tarnished plant bug	*Lygus lineolaris* (P. de B.)	Miridae (F)
Apple scab	*Venturia inaequalis* (Cke.) Wint.	Venturiaceae (F,L,W)
European red mite	*Panonychus ulmi* (Koch)	Tetranychidae (L)
Oblique-banded leafroller	*Choristoneura rosaceana* (Harr.)	Tortricidae (F,L)
**Secondary pests**		
European apple sawfly	*Hoplocampa testudinea* (Klug)	Tenthredinidae (F)
Eye-spotted budmoth	*Spilonota ocellana* (D. S.)	Tortricidae (F,L)
Apple plant bug	*Lygocoris communis* (Knight)	Miridae (F)
Rosy apple aphid	*Dysaphis plantaginea* (Pass.)	Aphididae (F)
Mullein bug	*Campylomma verbasci* (Meyer)	Miridae (F)
Speckled green fruitworm	*Orthosia hibisci* (Gn.)	Noctuidae (F)
Spotted tentiform leafminer	*Phyllonorycter blancardella* (F.)	Gracillairiidae (L)
Green apple aphid	*Aphis pomi* DeG.	Aphididae (L)
White apple leafhopper	*Typhlocyba pomaria* (McAtee)	Cicadellidae (L)
Oystershale scale	*Lepidosaphes ulmi* (L.)	Diaspididae (W)
Fruit-tree leafroller	*Archips argyrospila* (Wlk.)	Tortricidae (F)
Apple rust mite	*Aculus schlechtendali* (Nal.)	Tortricidae (F,L)
Red-banded leafroller	*Argyrotaenia velutinana* (Wlk.)	Tortricidae (F,L)
Buffalo treehopper	*Stictocephala bisonia* K. Y.	Membracidae (W)
Dogwood borer	*Synanthedon scitula* (Harr.)	Sesiidae (W)
Woolly apple aphid	*Eriosoma lanigerum* (Hausm.)	Aphididae (L,W)
**Minor pests**		
Apple grain aphid	*Rhopalosiphum fitchii* (Sand.)	Aphididae (L)
Lesser apple worm ^2^	*Grapholita prunivora* (Walsh)	Tortricidae (F)
Oriental fruit moth ^2^	*Grapholita molesta* (Busck)	Tortricidae (F,L)
Apple red bug	*Lygidea mendax* Reut.	Miridae (Fr)
Hawthorn dark bug	*Heterocordylus malinus* Reut.	Miridae (F)
Round-headed apple tree borer	*Saperda candida* (F.)	Cerambicidae (W)
Eastern tent caterpillar	*Malacosoma americanum* (F.)	Lasiocampidae (L)
Forest tent caterpillar	*Malacosoma disstria* Hbn.	Lasiocampidae (L)
Potato leafhopper	*Empoasca fabae* (Harris)	Cicadellidae (L)
Fall webworm	*Hyphantria cunea* (Drury)	Arctiidae (L)
Pale apple leafroller	*Pseudexentera mali* Free.	Tortricidae (F,L)
Brown marmorated stink bug	*Halyomorpha halys* (Stal)	Pentatomidae (F)
Green stink bug	*Acrosternum hilare* (Say)	Pentatomidae (F)
Brown stink bug	*Euschistus servus* (Say)	Pentatomidae (F)
Apple leafcurling midge	*Dasineura mali* (Kieffer)	Cecidomyidae (L)
European fruit scale	*Quadraspidiotus ostreaeformis* (Curt.)	Diaspididae (F,W)
Rose chafer	*Macrodactylus subspinosus* (F.)	Scarabeidae (F,L)
Japanese beetle	*Popillia japonica* (Newman)	Scarabeidae (F,L)

^1^ F=fruit; L= leaves; W= branches, trunk. ^2^ These tortricid species are commonly referred to as “internal lepidoptera” because of their feeding habits (see Table 2).

**Table 2:** Percent damage caused to fruit at harvest in participating orchards (1977–2001: n = 1000 fruits; 2002-2019: n = 500 fruits). Reference (insecticide-free) orchard located at Frelighsburg, Qc (Agriculture and Agri-Food Canada, 1977–2002) and Saint-Bruno-de-Montarville from 2003 to 2019. Commercial orchards located at Franklin, Hemmingford, Oka, Rougemont and Saint-Paul d’Abbotsford. PHASE1: average from 1977–2002. PHASE2: average from 2003–2019. OVERALL: average from 1977–2019).

	Internal lepidoptera				Codling Moth Only				Leafrollers				Oblique-Banded Leafroller Only				Plant Bugs				Tarnished Plant Bug Only				Plum Curculio				Apple Maggot				European Apple Sawfly				Apple Scab	
	Reference orchard	Commercial Orchards			Reference Orchard	Commercial Orchards			Reference Orchard	Commercial Orchards			Reference Orchard	Commercial Orchards			Reference Orchard	Commercial Orchards			Reference Orchard	Commercial Orchards			Reference Orchard	Commercial Orchards			Reference Orchard	Commercial Orchards			Reference Orchard	Commercial Orchards			Commercial Orchards	
1977	27.3	0.1	±0.0		18.7	0.0	±0.0		10.0	2.7	±1.3		1.9	0.0	±0.0		7.4	1.5	±0.5		2.5	1.2	±0.4		5.5	0.2	±0.1		3.7	0.0	±0.0		N/A	N/A			1.2	±0.5
1978	39.2	1.0	±0.3		25.4	0.1	±0.1		17.1	1.9	±1.2		1.1	0.0	±0.0		7.6	2.4	±0.7		1.5	1.8	±0.7		8.8	0.1	±0.0		2.5	0.3	±0.2		N/A	N/A			2.3	±1.4
1979	23.0	0.6	±0.2		19.7	0.1	±0.0		17.0	1.7	±0.8		2.2	0.3	±0.2		10.4	5.0	±0.9		5.5	3.9	±0.9		40.7	0.1	±0.1		10.3	0.0	±0.0		N/A	N/A			0.6	±0.5
1980	27.0	0.1	±0.1		22.4	0.1	±0.1		30.4	1.1	±0.2		0.2	0.1	±0.1		23.0	1.7	±0.3		5.9	0.9	±0.1		53.7	0.1	±0.0		31.8	0.2	±0.1		0.0	0.0	±0.0		2.0	±1.1
1981	32.8	0.0	±0.0		29.1	0.0	±0.0		13.9	0.9	±0.2		0.4	0.0	±0.0		34.3	2.7	±1.2		14.3	2.1	±1.2		85.3	0.9	±0.8		61.0	0.2	±0.1		1.2	0.7	±0.6		2.1	±1.0
1982	0.3	0.0	±0.0		0.3	0.0	±0.0		7.9	0.4	±0.1		0.1	0.0	±0.0		4.4	2.2	±0.4		1.0	1.5	±0.5		24.0	0.1	±0.1		0.9	0.0	±0.0		0.2	0.4	±0.3		3.6	±2.1
1983	9.9	0.0	±0.0		5.7	0.0	±0.0		5.4	0.5	±0.2		2.9	0.0	±0.0		13.5	2.3	±0.7		6.8	1.1	±0.2		37.0	0.1	±0.0		14.3	0.0	±0.0		0.4	0.1	±0.1		2.8	±1.4
1984	24.2	0.0	±0.0		18.9	0.0	±0.0		11.4	1.7	±1.5		6.6	1.4	±1.4		33.1	1.0	±0.3		6.2	0.4	±0.1		52.3	0.2	±0.1		10.6	0.0	±0.0		2.9	0.2	±0.1		0.8	±0.2
1985	23.2	0.1	±0.1		16.1	0.1	±0.1		27.9	2.2	±1.3		7.4	1.3	±1.2		35.3	1.4	±0.5		10.4	0.9	±0.4		59.2	0.0	±0.0		21.5	0.1	±0.0		0.9	0.1	±0.0		1.2	±0.8
1986	38.2	0.4	±0.4		34.4	0.1	±0.1		15.6	2.0	±1.1		2.0	0.1	±0.1		23.2	2.6	±1.4		6.3	1.6	±0.6		48.8	2.0	±1.1		51.6	0.5	±0.3		4.1	7.1	±3.0		3.2	±1.5
1987	48.2	2.3	±2.3		30.6	1.7	±1.7		20.3	1.0	±0.5		5.6	0.6	±0.6		35.7	4.8	±3.6		7.7	1.4	±1.3		84.6	0.2	±0.2		48.4	0.0	±0.0		0.7	0.0	±0.0		6.0	±3.1
1988	35.9	0.2	±0.2		22.0	0.0	±0.0		28.6	0.2	±0.1		5.0	0.0	±0.0		27.9	3.3	±1.3		13.3	1.9	±0.8		79.9	0.6	±0.6		23.7	0.7	±0.7		3.5	0.1	±0.1		2.1	±1.5
1989	9.7	0.0	±0.0		2.0	0.0	±0.0		40.0	0.7	±0.2		5.3	0.2	±0.2		33.7	3.9	±2.2		11.1	3.1	±1.8		39.4	0.1	±0.1		2.3	0.0	±0.0		7.4	0.0	±0.0		0.6	±0.2
1990	22.8	0.1	±0.1		19.0	0.0	±0.0		40.4	0.1	±0.1		N/A	0.0	±0.0		44.4	3.1	±1.0		4.4	2.5	±0.7		35.4	7.6	±7.4		81.6	0.0	±0.0		4.2	0.1	±0.1		1.3	±1.1
1991	N/A	N/A			23.0	0.1	±0.1		55.6	0.4	±0.1		3.2	0.0	±0.0		17.0	3.5	±2.1		5.2	2.8	±2.1		15.8	0.1	±0.1		46.0	0.0	±0.0		1.4	0.1	±0.1		0.2	±0.1
1992	N/A	N/A			16.6	0.0	±0.0		38.2	0.4	±0.2		5.6	0.0	±0.0		74.4	7.8	±3.4		12.6	7.5	±3.2		30.2	1.4	±1.3		28.2	0.3	±0.3		1.0	0.0	±0.0		0.1	±0.1
1993	N/A	N/A			58.4	0.0	±0.0		36.0	0.7	±0.3		20.4	0.0	±0.0		24.2	2.6	±1.0		4.6	2.2	±0.9		80.4	0.0	±0.0		49.0	0.1	±0.1		2.6	0.1	±0.1		0.2	±0.2
1994	43.2	0.0	±0.0		43.2	0.0	±0.0		17.8	0.8	±0.5		4.4	0.1	±0.1		23.4	1.1	±0.4		3.4	0.7	±0.4		86.0	0.3	±0.2		55.8	0.0	±0.0		1.2	0.0	±0.0		0.2	±0.2
1995	38.0	0.0	±0.0		38.0	0.0	±0.0		55.4	0.2	±0.1		4.8	0.0	±0.0		45.2	3.2	±2.4		3.2	2.6	±2.1		88.2	0.2	±0.1		98.4	0.0	±0.0		1.0	0.7	±0.7		1.5	±0.9
1996	10.2	0.0	±0.0		10.2	0.0	±0.0		47.4	0.9	±0.3		0.6	0.1	±0.1		25.4	1.5	±0.5		3.6	1.0	±0.3		39.4	0.3	±0.2		90.0	0.0	±0.0		1.4	0.0	±0.0		1.2	±1.0
1997	15.2	0.0	±0.0		15.2	0.0	±0.0		64.6	1.7	±1.3		1.0	0.1	±0.0		17.6	0.4	±0.1		3.0	0.2	±0.1		86.2	0.0	±0.0		96.8	0.0	±0.0		1.8	0.0	±0.0		0.3	±0.2
1998	16.8	0.0	±0.0		16.8	0.0	±0.0		31.4	1.2	±1.0		1.0	1.1	±1.0		12.0	3.3	±0.9		6.2	2.9	±0.8		48.0	0.0	±0.0		94.6	0.0	±0.0		7.2	0.5	±0.2		0.4	±0.3
1999	N/A	0.1	±0.1		N/A	0.1	±0.1		N/A	2.4	±1.0		N/A	0.8	±0.4		N/A	0.7	±0.4		N/A	0.3	±0.2		N/A	0.1	±0.1		N/A	0.0	±0.0		N/A	2.2	±1.9		0.1	±0.1
2000	27.2	0.0	±0.0		27.2	0.0	±0.0		62.0	1.8	±0.5		4.8	0.7	±0.5		30.4	1.6	±0.5		11.2	1.4	±0.4		88.8	0.3	±0.2		86.8	0.0	±0.0		7.8	0.1	±0.0		1.3	±1.1
2001	22.2	0.1	±0.0		22.2	0.1	±0.0		69.6	3.5	±0.8		12.6	1.2	±0.7		24.4	4.5	±2.4		9.4	3.7	±2.0		89.6	0.3	±0.1		89.8	0.0	±0.0		9.4	0.5	±0.3		0.2	±0.1
2002	39.0	0.0	±0.0		39.0	0.0	±0.0		56.0	2.6	±1.3		9.4	0.5	±0.4		18.4	0.9	±0.5		4.8	0.7	±0.4		82.2	0.1	±0.1		93.2	0.0	±0.0		4.8	0.6	±0.3		0.3	±0.1
**PHASE1**	**26.1**	**0.2**	**±0.1**		**23.0**	**0.1**	**±0.1**		**32.8**	**1.3**	**±0.2**		**4.5**	**0.3**	**±0.1**		**25.9**	**2.6**	**±0.3**		**6.6**	**1.9**	**±0.3**		**55.6**	**0.6**	**±0.3**		**47.7**	**0.1**	**±0.0**		**3.0**	**0.6**	**±0.3**		**1.4**	**±0.3**
																																						
2003	2.0	0.4	±0.3		2.0	0.4	±0.3		10.0	2.2	±0.5		1.0	0.7	±0.3		9.0	2.2	±0.6		6.0	1.6	±0.2		24.0	0.9	±0.5		1.0	0.0	±0.0		4.0	0.1	±0.0		0.4	±0.3
2004	N/A	0.4	±0.3		N/A	0.4	±0.3		N/A	2.6	±0.8		N/A	0.3	±0.1		N/A	1.5	±0.5		N/A	0.6	±0.2		N/A	0.1	±0.0		N/A	0.0	±0.0		N/A	0.5	±0.4		0.2	±0.1
2005	N/A	0.5	±0.3		N/A	0.5	±0.3		N/A	1.5	±0.5		N/A	0.1	±0.1		N/A	0.8	±0.4		N/A	0.6	±0.3		N/A	0.6	±0.6		N/A	0.0	±0.0		N/A	0.4	±0.3		1.0	±0.9
2006	N/A	0.9	±0.6		N/A	0.9	±0.6		N/A	1.5	±0.5		N/A	0.3	±0.2		N/A	0.9	±0.2		N/A	0.8	±0.2		N/A	0.0	±0.0		N/A	0.0	±0.0		N/A	0.4	±0.2		2.8	±2.3
2007	N/A	1.1	±0.6		N/A	1.1	±0.6		N/A	0.9	±0.3		N/A	0.3	±0.1		N/A	2.9	±1.0		N/A	1.5	±0.2		N/A	0.2	±0.2		N/A	0.0	±0.0		N/A	0.3	±0.2		4.3	±2.9
2008	N/A	0.1	±0.1		N/A	0.1	±0.1		N/A	1.0	±0.6		N/A	0.0	±0.0		N/A	0.8	±0.2		N/A	0.5	±0.2		N/A	0.3	±0.2		N/A	0.2	±0.1		N/A	0.9	±0.4		3.5	±3.0
2009	N/A	0.2	±0.1		N/A	0.2	±0.1		N/A	1.2	±0.4		N/A	0.1	±0.1		N/A	1.4	±0.6		N/A	1.1	±0.6		N/A	0.1	±0.1		N/A	0.0	±0.0		N/A	0.6	±0.4		0.8	±0.6
2010	N/A	1.2	±1.0		N/A	1.2	±1.0		N/A	0.1	±0.1		N/A	0.1	±0.1		N/A	0.7	±0.4		N/A	0.7	±0.4		N/A	0.1	±0.1		N/A	0.4	±0.4		N/A	0.5	±0.2		0.8	±0.8
2011	9.1	0.4	±0.3		9.1	0.4	±0.3		5.1	0.1	±0.1		0.3	0.1	±0.1		5.3	2.3	±0.8		3.3	2.2	±0.8		8.8	0.0	±0.0		93.0	0.3	±0.3		2.9	0.6	±0.5		1.7	±1.5
2012	11.7	1.1	±0.2		11.7	1.1	±0.2		3.7	0.2	±0.1		0.0	0.1	±0.1		2.9	1.7	±0.6		0.3	1.2	±0.4		16.3	0.1	±0.1		72.7	0.3	±0.1		8.0	0.7	±0.3		1.2	±0.6
2013	13.0	0.6	±0.3		13.0	0.6	±0.3		4.0	0.2	±0.1		0.0	0.1	±0.1		7.3	3.0	±0.5		4.3	1.8	±0.7		16.0	0.3	±0.2		60.3	0.2	±0.1		1.3	0.4	±0.3		0.6	±0.6
2014	27.7	1.0	±0.2		27.7	1.0	±0.2		15.0	0.2	±0.2		0.3	0.0	±0.0		9.6	1.6	±0.7		7.0	1.3	±0.6		23.3	0.9	±0.6		91.7	0.6	±0.5		1.0	0.1	±0.1		0.1	±0.1
2015	3.3	0.1	±0.1		3.3	0.1	±0.1		15.6	0.0	±0.0		0.3	0.0	±0.0		11.6	2.3	±0.9		6.6	2.0	±0.9		16.3	0.3	±0.2		73.4	0.4	±0.4		5.3	0.6	±0.4		4.1	±4.1
2016	4.7	0.2	±0.1		4.7	0.2	±0.1		14.1	0.2	±0.2		0.7	0.1	±0.1		10.6	3.1	±1.0		6.3	2.7	±1.1		78.7	0.2	±0.2		59.3	0.9	±0.4		1.7	0.2	±0.1		0.5	±0.5
2017	10.0	0.4	±0.4		10.0	0.4	±0.4		13.7	0.2	±0.2		0.7	0.0	±0.0		20.0	2.2	±1.0		12.7	1.7	±0.7		31.3	0.4	±0.2		22.7	0.2	±0.2		0.3	0.1	±0.0		2.7	±1.6
2018	22.7	1.0	±0.6		22.0	1.0	±0.6		12.3	0.4	±0.3		0.0	0.3	±0.3		15.0	1.0	±0.5		9.3	0.9	±0.5		60.3	0.0	±0.0		66.3	0.3	±0.2		2.0	0.1	±0.1		0.1	±0.1
2019	6.7	1.7	±0.7		6.7	1.7	±0.7		10.3	0.3	±0.2		0.3	0.2	±0.2		6.3	1.2	±0.6		2.3	0.6	±0.3		10.3	0.6	±0.4		16.3	0.8	±0.6		0.0	0.0	±0.0		0.0	±0.0
**PHASE2**	**11.1**	**0.7**	**±0.1**		**11.0**	**0.7**	**±0.1**		**10.4**	**0.8**	**±0.2**		**0.4**	**0.2**	**±0.0**		**9.8**	**1.8**	**±0.2**		**5.8**	**1.3**	**±0.2**		**28.5**	**0.3**	**±0.1**		**55.7**	**0.3**	**±0.1**		**2.7**	**0.4**	**±0.1**		**1.5**	**±0.4**
																																						
**OVERALL**	**21.4**	**0.4**	**±0.1**		**19.6**	**0.3**	**±0.1**		**26.4**	**1.1**	**±0.1**		**3.3**	**0.3**	**±0.1**		**21.3**	**2.3**	**±0.2**		**6.3**	**1.7**	**±0.2**		**47.8**	**0.5**	**±0.2**		**50.0**	**0.2**	**±0.0**		**2.9**	**0.5**	**±0.2**		**1.4**	**±0.2**

**Table 3:** Average number and cost (± standard error) of pesticide sprays per season in participating orchards. Commercial orchards located at Franklin, Hemmingford, Oka and Rougemont from 1978 to 2001 (Phase 1) and at Dunham, Franklin, Hemmingford, Oka, Saint-Joseph, Compton and Sainte-Famille from 2002 to 2019 (Phase 2). Phase 1: average from 1977–2001. Phase 2: average from 2002–2019. OVERALL: average from 1977–2019. Costs calculated from average price of pesticides published annually by the Apple Pest Warning Service (Table S1). N/A data not available.

	Insecticides					Acaricides					Fungicides ^1^			
	Number ^2^		Cost ^2^			Number ^2^		Cost ^3^			Number ^2^		Cost ^3^	
1978	4.3	±0.6	254.87	±33.1		1.5	±0.3	160.59	±27.2		11.8	±1.4	490.77	±56.7
1979	5.3	±1.1	270.66	±30.2		1.3	±0.3	142.94	±10.8		10.0	±2.0	420.04	±53.5
1980	5.0	±1.1	333.05	±72.6		1.5	±0.5	206.55	±21.6		8.8	±1.7	522.11	±75.0
1981	2.7	±0.7	206.88	±70.9		1.1	±0.3	162.31	±49.6		9.9	±1.2	550.8	±164.7
1982	3.7	±0.4	311.56	±60.7		1.1	±0.3	153.05	±35.2		12.3	±1.2	648.76	±106.2
1983	3.3	±0.8	270.62	±77.0		1.5	±0.3	224.43	±34.8		14.6	±1.1	740.7	±71.5
1984	4.2	±0.0	376.18	±17.2		1.1	±0.2	223.21	±48.3		8.8	±0.5	594.2	±87.6
1985	3.5	±1.3	306.42	±95.7		1.5	±0.7	245.22	±96.7		10.6	±0.8	742.31	±73.2
1986	2.9	±0.4	242.00	±56.1		0.9	±0.1	118.22	±43.1		13	±0.3	749.38	±45.8
1987	2.6	±1.6	199.73	±154.6		2.5	±1.5	327.57	±186.0		15	±2.7	797.27	±225.8
1988	2.7	±0.1	191.93	±32.6		1.5	±0.3	169.98	±40.0		9.9	±0.7	597.19	±82.2
1989	3	±0.5	219.28	±46.0		1.6	±0.7	247.43	±106.4		7.4	±1.7	396.66	±106.7
1990	4	±1.0	313.78	±68.5		2.4	±0.8	328.77	±144.7		9.3	±1.2	576.86	±97.4
1991	2.8	±0.2	204.66	±18.8		2.2	±0.4	349.00	±68.0		10.4	±1.6	614.93	±92.5
1992	N/A		285.44	±58.0		2.1	±0.2	352.13	±49.4		N/A		852.39	±172.7
1993	3.2	±0.3	312.73	±40.2		1.5	±0.1	281.67	±35.6		6.8	±0.8	598.35	±106.3
1994	3	±0.4	207.06	±46.9		1.7	±0.1	238.34	±29.6		5.7	±0.5	480.5	±55.9
1995	2.6	±0.3	181.52	±19.2		2.5	±0.3	470.32	±59.2		8.8	±1.3	797.31	±208.6
1996	3.1	±0.6	189.09	±36.8		2.2	±0.2	353.89	±50.4		10.5	±1.8	723.36	±132.5
1997	3.4	±0.6	205.10	±90.8		1.3	±0.3	393.33	±123.8		10.2	±2.4	693.6	±163.4
1998	3.5	±0.7	215.13	±30.0		1.1	±0.2	259.09	±91.6		6.6	±1.1	325.09	±50.6
1999	4.5	±0.1	356.84	±51.0		1.7	±0.1	394.58	±57.4		7.5	±1.2	454.18	±65.4
2000	4.6	±0.8	401.38	±86.1		2	±0.3	464.65	±99.2		9.6	±1.7	631.16	±58.5
2001	5.4	±0.8	362.70	±31.0		2.2	±0.3	604.00	±98.2		16.0	±2.9	847.28	±149.6
Phase 1	3.6	±0.2	267.44	±13.7		1.67	±0.1	286.30	±24.7		10.2	±0.6	618.55	±29.8
														
2002	3.6	±0.6	299.86	±54.3		1.3	±0.3	310.07	±58.2		18.6	±2.7	1107.61	±106.9
2003	3.2	±0.7	260.62	±58.5		1.7	±0.4	333.43	±95.4		16.8	±2.4	971.85	±99.5
2004	3.6	±0.6	245.59	±56.6		1.0	±0.2	174.90	±42.0		15.9	±2.1	855.43	±91.0
2005	3.5	±0.7	240.82	±42.8		1.6	±0.3	361.98	±64.0		14.1	±2.0	732.15	±96.8
2006	3.2	±0.8	258.32	±52.1		1.2	±0.2	214.76	±45.7		16.4	±2.9	799.42	±135.3
2007	4.9	±1.4	327.41	±79.7		1.2	±0.3	259.86	±70.9		14.5	±1.5	762.96	±104.6
2008	4.1	±0.7	328.84	±55.2		1.6	±0.3	313.92	±62.6		14.3	±1.1	866.18	±108.6
2009	3.6	±0.6	368.03	±55.7		1.6	±0.2	419.82	±65.0		16.8	±1.4	1565.96	±226.5
2010	4.8	±0.5	422.97	±39.2		1.4	±0.3	302.05	±76.6		14.8	±1.5	1040.86	±199.2
2011	4.1	±0.7	355.19	±60.0		1.9	±0.3	394.57	±72.6		16.5	±1.9	1213.42	±228.6
2012	4.1	±0.4	374.43	±44.3		1.4	±0.2	295.53	±45.1		17.9	±1.7	1339.37	±119.9
2013	5.1	±0.7	506.33	±106.3		1.2	±0.2	224.54	±36.9		17.6	±1.6	1321.09	±117.8
2014	4.7	±0.7	540.13	±128.1		1.6	±0.2	302.26	±52.0		12.8	±2.6	863.36	±197.9
2015	5.1	±0.9	573.48	±93.6		1.8	±0.3	306.24	±40.6		15.7	±1.9	1050.60	±186.4
2016	4.4	±0.8	658.51	±91.3		1.3	±0.2	275.43	±51.1		14.5	±2.8	1037.99	±203.5
2017	4.5	±0.6	667.77	±94.9		1.2	±0.2	247.28	±43.8		17.7	±1.5	1009.71	±120.0
2018	4.9	±1.7	782.67	±134.5		1.3	±0.3	280.73	±59.5		13.9	±1.5	781.82	±93.8
2019	4.5	±0.9	733.76	±112.0		1.2	±0.2	213.86	±39.8		14.9	±2.0	787.62	±121.0
Phase 2	4.2	±0.2	441.40	±42.0		1.4	±0.1	290.60	±15.0		15.8	±0.4	1006.00	±54.8
OVERALL	3.9	±0.1	342.00	±23.5		1.6	±0.1	288.20	±15.4		12.6	±0.6	784.60	±41.4

^1^ including bactericides used against some diseases; ^2^ each application done on the whole orchard using the recommended dose correspond to one application. Sprays applied as mixtures (e.g., fungicide and insecticide) were assigned to their corresponding categories. ^3^ pesticides only, expressed in 2019 constant CAD, based on agricultural price indexes for Quebec, Fresh Fruit category, published annually by Statistics Canada.

## Data Availability

The data are not publicly available due to privacy concerns; requests will be fulfilled within the limits of the relevant privacy acts of the government of Québec (Loi sur la protection des renseignements personnels dans le secteur privé and Loi sur l’accès aux documents des organismes publics et sur la protection des renseignements personnels).

## Supplementary Materials

The following are available online: https://www.mdpi.com/2075-4450/12/3/197/s1. Table S1: Data source for pesticide costs, 1978-2019; Figure S2: Average trap captures per year over the course of the study for insect pests followed in commercial orchards. Table S3: Acaricides, Insecticides and fungicides recommended for use in Quebec apple orchards between 1977 and 2019; Table S4: Linear regressions of the number and cost of pesticides sprays by season against time.
